# Clinical-Pathological Conference Series from the Medical University of Graz: Case No 183: A 57-year-old farmer with fever, cough, maculopapular rash and bicytopenia

**DOI:** 10.1007/s00508-025-02586-8

**Published:** 2025-09-23

**Authors:** Elisabeth Fabian, Maximilian Gornicec, Nikolaus Kneidinger, Albert Wölfler, Florentine Moazedi-Fürst, Marijan Puseljic, Michael Fuchsjäger, Robert Krause, Guenter J. Krejs

**Affiliations:** 1 Department of Internal Medicine II, University Hospital Krems, Karl Landsteiner University of Health Sciences, Krems on the Danube, Austria; 2 Division of Infectious Diseases, Department of Internal Medicine, Medical University of Graz, Graz, Austria; 3 Division of Respiratory Medicine, Department of Internal Medicine, Lung Research Cluster, Medical University of Graz, Graz, Austria; 4 Division of Hematology, Department of Internal Medicine, Medical University of Graz, Graz, Austria; 5 Division of Rheumatology, Department of Internal Medicine, Medical University of Graz, Graz, Austria; 6 Department of General Radiology, Medical University of Graz, Graz, Austria; 7 Division of Gastroenterology and Hepatology, Department of Internal Medicine, Medical University of Graz, Auenbruggerplatz 15, 8036 Graz, Austria

**Keywords:** Autoinflammation, Anemia, Arthralgia, Pulmonary infiltrates, VEXAS syndrome

## Presentation of case

### Dr. M. Gornicec:

The patient presented to the outpatient clinic of another hospital with fever (39.7 °C) and cough one week prior to admission to our facility. He had a 1-year history of bicytopenia (leukocytes 2.5 10^9^/L [normal: 4.4–11.3 10^9^/L], red cells 3.5 10^12^/L [normal: 4.5–5.9 10^12^/L]) with follow-up tests carried out every four weeks. A bone marrow biopsy performed nine months earlier, including cytogenetics and molecular genetic testing for the presence of recurrent mutations associated with myeloid neoplasms was unremarkable. Genetic testing for monoclonal rearrangement of the T‑cell receptor genes was also unremarkable. Due to migratory polyarthralgia affecting large and small joints, the patient was seen by a rheumatologist. There was no evidence of joint swelling, therefore the cause of the complaints was eventually narrowed down to neuropathic arthropathy. This was confirmed by a nerve conduction study on the lower legs (sensory axonal neuropathy). With the recent suspicion of Lyme disease (IgG antibodies to *Borrelia burgdorferi* positive, IgM antibodies negative), the patient was on antibiotics (clindamycin, metronidazole and clarithromycin) prescribed by his general practitioner. A comprehensive search for an infectious cause of his fever and cough was carried out on an outpatient basis. While the PCR test for severe acute respiratory syndrome coronavirus 2 (SARS-CoV2) was negative, it was positive for influenza A. The chest radiograph showed peribronchial wall thickening but was otherwise unremarkable; a computed tomography (CT) of the chest was also unremarkable apart from revealing an enlarged axillary lymph node (short axis diameter: 1.5 cm). The patient was prescribed Tamiflu® (oseltamivir, 75 mg b.i.d. for five days) and paracetamol as needed for use at home.

In view of persistent fever and cough, and the new onset of an accentuated confluent maculopapular rash, the patient was admitted to the University Medical Center Graz. An influenza PCR test was now negative. There were no abnormalities on physical examination apart from the rash. The ECG showed sinus rhythm and was unremarkable. Laboratory tests: C‑reactive protein (CRP) 158 mg/L (normal: <5.0 mg/L, in further course up to 350 mg/L), procalcitonin 0.15 ng/mL (normal: <0.5 ng/mL, in further course up to 4.5 ng/mL), soluble interleukin (IL)-2 receptor 7924.3 pg/mL (normal: 458.0–1997.0 pg/mL), ferritin 1276 ng/mL (normal: 18–360 ng/mL), red cells 3.5 10^12^/L (normal: 4.1–6.8 10^12^/L), hemoglobin 10.8 g/dL (normal: 13.0–17.5 g/dL), hematocrit 31.2% (normal: 39.5–50.0%), mean corpuscular volume (MCV) 88.9 fL (normal: 80.0–98.0 fL), platelets 313 10^9^/L (normal: 140–440 10^9^/L), differential blood count: lymphocytes 13% (normal: 20–44%), otherwise unremarkable, gamma-glutamyl transferase (GGT) 128 U/L (normal: <55 U/L), aspartate aminotransferase (AST) 58 U/L (normal: <50 U/L), alanine aminotransferase (ALT) 68 U/L (normal: <50 U/L), lactate dehydrogenase 258 U/L (normal: 120–240 U/L), fasting glucose 116 mg/dL, lactate 0.8 mmol/L (normal: 0.5–2.2 mmol/L), prothrombin time 57% (normal: 70–100%). Antinuclear antibodies (ANA) and antineutrophil cytoplasmic antibodies (ANCA) screened negative.

Antibiotic therapy with ceftriaxone and doxycycline had no clinical or laboratory effect. The rash was assessed by dermatologists three times and was considered to be either of viral (measles PCR negative) or drug-induced etiology (most likely due to clindamycin or oseltamivir), or a dermatosis akin to Sweet syndrome; however, histologic examination of a punch biopsy skin sample did not show changes typical for Sweet syndrome. A follow-up chest radiograph again showed peribronchial wall thickening and the follow-up CT of the chest was again unremarkable except for the previously noted axillary lymph node enlargement (1.5 cm). Abdominal sonography showed splenomegaly (15.5 cm) and a double-barrel shotgun sign in the left lobe of the liver. Abdominal CT was unremarkable except for splenomegaly; magnetic resonance cholangiopancreatography was also unremarkable. An 18F-FDG PET-CT revealed diffuse tracer uptake in the posterior basal lung segments consistent with gravity-induced fluid accumulation with questionable additional inflammatory signs. Furthermore, the scan showed reactive lymph nodes in the left axilla, right hilar and parahilar, and parailiac and parainguinal, bilaterally and symmetrically. Further, there was a homogeneously increased tracer uptake in the enlarged spleen, corresponding to reactive changes of the spleen and signs of central bone marrow activation. A cranial CT showed an inconspicuous neurocranium and regularly aerated paranasal sinuses. A surgical biopsy of an axillary lymph node revealed only fatty tissue. Transthoracic echocardiography was unremarkable. In view of lacrimation and pain in the left eye, the ophthalmologist diagnosed iridocyclitis.

A further comprehensive work-up for underlying infectious diseases revealed negative results for blood and urine cultures, as well as negative serology and/or PCR tests for HIV, parvovirus B‑19, HSV, CMV, EBV, viral hepatitis, Coxsackie virus, Brucella spp., *Coxiella burnetii, Rickettsia conorii, Bartonella henselae, Treponema pallidum,* Leptospira spp. and Mycoplasma spp.; urinary antigen tests for legionella and pneumococcus as well as QuantiFERON^®^ TB (QIAGEN, Hilden, Germany) tests were also negative. A positive toxoplasma serology was not considered causative in this case. The follow-up ANA and ANCA screening was again negative.

A therapeutic trial with corticosteroids resulted in a rapid improvement of symptoms, a marked reduction of the rash and a very rapid regression of parameters of inflammation.

A diagnostic test was performed.

## Differential diagnosis

### Dr. N. Kneidinger:

This is an interesting case of a farmer who presented with fever, cough, maculopapular rash and bicytopenia. Focusing on the symptoms of fever and cough, and in view of the fact that the patient is a farmer, the list of differential diagnoses includes nonspecific infections of the airways, infection with *Coxiella burnetii* (Q fever) and hypersensitivity pneumonitis. *Coxiella burnetii* is an intracellular bacterium naturally infecting animals, such as goats, sheep and cows. The most common mode of transmission to humans is inhalation of *Coxiella burnetii* that has been excreted by animals, especially livestock (i.e. dust that has been contaminated by infected animal feces, urine, milk and birth products); however, only about half of people who are infected become ill. The illness typically develops 2–3 weeks after being exposed to the bacteria and presents with flu-like symptoms including fever, chills, fatigue and myalgia. Some people also develop chronic Q fever after they are infected [1, 2]. The rate of symptomatic Q fever in North America and the European Union (EU) is relatively low with an average annual incidence from 2008–2017 of 0.045 cases per 100,000 persons in the USA [3] and an incidence of 0.2 cases per 100,000 persons in the EU [4]. Although infection with *Coxiella burnetii* is a potential diagnosis due to the clinical presentation and occupational history of the patient, it can be ruled out because of negative microbiology findings for this pathogen.

Hypersensitivity pneumonitis is a complex syndrome of varying intensity, clinical presentation and natural history. It represents an immunologic reaction to an inhaled agent, particularly an organic antigen such as agricultural dusts, bioaerosols and certain reactive chemical species, occurring within the pulmonary parenchyma [5]; however, an essentially unremarkable CT of the chest and the clinical course of the discussed patient makes this diagnosis unlikely.

Bicytopenia has been known for about one year in this patient and laboratory monitoring has shown a stable situation. This finding may be indicative of a bone marrow disease, such as leukemia, myelodysplastic syndromes/neoplasms (MDS), osteomyelofibrosis or aplastic anemia. In addition, toxic causes (e.g. alcohol), chronic infections (e.g. viral hepatitis or HIV), hemolytic anemia, autoimmune disorders (e.g. systemic lupus erythematosus) and micronutrient deficiencies (e.g. vitamin B_12_, folate, iron) should also be considered; however, a normal bone marrow biopsy, an unremarkable history regarding intoxication and available laboratory results rule out the mentioned diagnoses in the discussed patient.

Migratory pain affecting large and small joints was most consistent with arthralgia of neuropathic etiology in this case. Possible underlying causes of neuropathy include, among others, metabolic diseases such as diabetes mellitus, chronic kidney disease, deficiency in B vitamins, alcohol use disorder and autoimmune diseases. Further, some infectious diseases such as Lyme disease (initially suspected by the patient’s general practitioner although IgM antibodies to *Borrelia burgdorferi* were negative, only IgG antibodies were positive) or influenza (for which our patient tested positive) frequently involve the joints. In summary, joint complaints along with bicytopenia could indeed occur secondary to influenza, but as both had existed for several months in the discussed patient, this hypothesis seems rather unlikely.

As the respiratory complaints and fever persisted, and the patient additionally developed a maculopapular rash, he was admitted for inpatient diagnostic work-up and treatment. As far as the rash is concerned, the possible underlying diagnoses include drug hypersensitivity syndrome or drug rash with eosinophilia and systemic symptoms (DRESS), infectious diseases (e.g. scarlet fever, measles), autoimmune diseases, sarcoidosis, dermatomyositis, hemophagocytic syndrome and acute febrile neutrophilic dermatosis; however, these diagnoses can be taken off the list of differential diagnoses because it seems quite unlikely that the medication taken by the patient had induced DRESS, and he further tested negative for measles, ANA and ANCA.

Diagnostic imaging including CT, abdominal sonography and 18F-FDG PET-CT showed splenomegaly, lymphadenopathy with reactive lymph nodes in the left axilla, the hilar and parahilar area on the right, and in the parailiac and parainguinal areas bilaterally. It also revealed signs of central bone marrow activation but was otherwise unremarkable. Indeed, these findings are rather nonspecific and point to a certain systemic proinflammatory situation. The finding of iridocyclitis in the left eye is suggested to be most likely of autoimmune or infectious origin; however, an additional comprehensive work-up for underlying infectious diseases was negative. Moreover, negative laboratory findings excluded autoimmunity as a cause of iridocyclitis.

A therapeutic trial with corticosteroids led to a rapid improvement of symptoms including the rash and a very rapid regression of parameters of inflammation.

In summary, this patient clearly suffered from a hyperinflammatory syndrome with multiorgan manifestation. Such a hyperinflammatory situation can be associated with certain autoimmune diseases or may occur secondary to a hematologic disease; however, based on the particular constellation of laboratory and radiologic findings in this case, neither seem to apply. When this hyperinflammatory situation suggesting an autoinflammatory disease is further compounded by the fact that the patient is a white man who presented with joint complaints and a hematologic condition (bicytopenia), the most likely diagnosis in this case is vacuoles, E1 enzyme, X‑linked, autoinflammatory, somatic (VEXAS) syndrome. This syndrome, first described in 2020 [6], is an adult onset autoinflammatory syndrome characterized by somatic mutations in the *UBA1* gene and is considered to be the prototype of hemato-inflammatory diseases. Mutations in the chromosome X‑linked *UBA1* gene are monogenic, somatic and restricted to cells of the myeloid and erythroid lineage [7]. VEXAS has been mainly reported in men but rarely occurs also in women [8]. Patients often present with a combination of inflammatory, rheumatologic and hematologic conditions. The most common manifestations include skin lesions, fever, chondritis, arthritis, pulmonary infiltrates, anemia, thrombocytopenia and venous thrombosis [9]. Only genetic testing can unequivocally confirm this diagnosis.

## Dr. N. Kneidinger’s diagnosis

VEXAS syndrome

## Discussion of case

### Dr. M. Gornicec:

As summarized by Dr. Kneidinger, this case was challenging because the patient’s multiple clinical manifestations were mimicking a variety of diseases; however, the progressive and refractory inflammatory condition, involving the lungs and the skin, along with clinical features bridging rheumatologic and hematologic symptoms, led to VEXAS syndrome as our working diagnosis. This was finally confirmed by genetic testing that revealed a mutation in the *UBA1* gene (p.M41Thr, VAF [variant allele fraction]: 49.29 and 49.71%). VEXAS defines a new disease category, i.e. the hemato-inflammatory disorders triggered by somatic mutations restricted to blood but causing systemic inflammation with multiorgan involvement and aberrant bone marrow status [10].

Treatment of VEXAS involves glucocorticoids and other immunosuppressants. In the discussed patient administration of glucocorticoids rapidly led to the improvement of symptoms including skin lesions and the regression of inflammation parameters. Therapy was tapered after one week. Currently, the patient is under therapy with the Janus kinase inhibitor ruxolitinib and prednisone 7.5 mg daily. His clinical condition is stable with remission of all inflammatory symptoms.

### Dr. A. Wölfler:

VEXAS is an acronym based on the key features of the syndrome, i.e. vacuoles, E1 enzyme, X‑linked, autoinflammation, somatic. The syndrome was first identified in 2020 by Beck et al. and is caused by mutations in the *UBA1* gene [11]. Mutations were identified as monogenic, somatic and restricted to cells of the myeloid and erythroid lineage [6]. The *UBA1* gene encodes an E1 enzyme, which plays a fundamental role for the ubiquitination pathway [12]. The ubiquitin-activating enzyme (E1) is considered as the main initiator of activation, conjugation and binding of ubiquitin to protein substrates for degradation by the ubiquitin-proteasome system [13, 14]. Its functionality is indispensable for the ubiquitination of many proteins and is thus pivotal for a variety of cellular processes. The *UBA1* is expressed in a nuclear isoform (UBA1a) and a cytoplasmic isoform (UBA1b) [15]. The most frequent mutation in VEXAS is found in codon 41 of *UBA1 *where translation of UBA1b is initiated. It causes substitution of methionine: p.Met41Thr (c.122T>C), p.Met41Val (c121A>G) and p.Met41Leu (c121A>C). Mutation in p.Met41 leads to translation initiation to shift to p.Met67, which subsequently results in a decrease in the cytoplasmic UBA1b isoform and an increased production of the new and dysfunctional UBA1 cytoplasmic isoform, UBA1c. [6]. Depending on the mutation, the translation form M41 can still occur with lower efficiency (M41L and M41T maintain 10–15% of wild-type protein level; M41V only 5%) [16]. Other *UBA1* mutations not affecting M41 include splicing variants, which lead to an in-frame deletion of short exon segments containing M41 [17–19] and mutations affecting functional sites in the region shared by UBA1a and UBA1b isoforms, resulting in a partial loss of function of both the nuclear and the cytoplasmic isoforms without the formation of the UBA1c isoform [18, 20, 21]. The existence of non-M41 mutations suggests that VEXAS syndrome is caused by the decrease of ubiquitin activation in the cytoplasm and is not due to the generation of UBA1c or the impairment of UBA1a [11].

*UBA1 *is an X‑linked gene located on chromosome Xp11.23 that escapes X inactivation; thus, VEXAS syndrome occurs almost exclusively in men. Rarely, it also affects women with constitutional 45,X karyotype (monosomy X, Turner syndrome) [8] and possibly with uniparental disomy, biallelic mutation or skewed X inactivation [22]. Acquired monosomy X due to mosaicism in the X chromosome is the most commonly reported reason underlying a diagnosis of VEXAS in women [8, 56]. Mosaicism of the X chromosome is an age-related phenomenon and preferentially affects the inactivated X chromosome. It has been shown to occur in 0.11% of 50-year-old women with an increase to 0.45% in 75-year-old women [23].

Ubiquitination impairment seen in VEXAS syndrome leads to an upregulation of the unfolded protein response, probably due to the decrease in the efficiency of endoplasmic reticulum-associated degradation and the consequent accumulation of misfolded proteins [24]. Unfolded protein response can trigger inflammation via many different mechanisms (e.g. NF-kB pathway, activation of the inflammasome) and is associated with multiple autoimmune and autoinflammatory diseases [25, 26]. Patients with VEXAS syndrome show high levels of proinflammatory cytokines (e.g. IL-1ß, IL-18) and CRP; neutrophils spontaneously release the inflammogenic neutrophil extracellular trap [6, 11] and monocytes aberrantly express chemokine receptors that may facilitate migration of immune cells and inflammosome activation in the skin [27].

Erythropoiesis, megakaryopoiesis and lymphopoiesis are all regulated by ubiquitination. Thus, *UBA1* loss-of-function mutations in VEXAS syndrome have a noticeable effect on hematopoietic stem and progenitor cells. Bone marrow examination typically shows a hyperplastic bone marrow with increase in myeloid progenitors and decrease in erythroid progenitors. Both progenitor cell types show characteristic vacuoles, which seem to be autophagic vacuoles indicating stress [28]. In general, vacuoles in myeloid and erythroid precursor cells are rare. Although they occur in patients with VEXAS (predominantly in promyelocytes, myelocytes, erythroid precursors and blasts in the marrow), they are not specific for the syndrome and can also be found in patients with alcohol use disorder [29], copper and zinc deficiency [30–32] or myeloid neoplasms [33]. In the discussed patient, only some isolated small vacuoles were found in a second, retrospective examination of the bone marrow. Further, megakaryocytes also show characteristic dysplasia [34]. Patients with VEXAS syndrome typically present with cytopenia, including red cells (macrocytic anemia as the predominant cytopenia), platelets, neutrophils, monocytes and lymphocytes, especially B cells [16].

A substantial portion of these patients develop hematologic malignancies, including MDS and, in rare cases, multiple myeloma. Despite the increased risk of acute myeloid leukemia (AML) in MDS patients [35], progression to AML is extremely rare in patients with VEXAS-MDS [36].

**Fig. 1:**
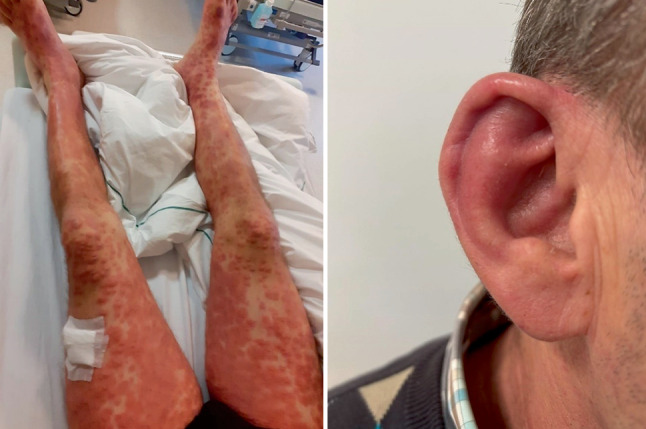
Neutrophilic dermatosis on the legs (*left image*) and chondritis of the ear (*right image*) of the discussed patient with VEXAS syndrome

Besides the described hematologic conditions, clinical features of VEXAS frequently include recurrent fever, systemic inflammation involving the skin (neutrophilic dermatosis, Fig. 1), lungs, blood vessels (medium vessel or leukocytoclastic vasculitis) and cartilage (e.g. auricular chondritis, Fig. 1) [7]. The latter was also present in our patient but was not mentioned in the case protocol. As in our patient, skin manifestations have been reported in up to 84% of VEXAS syndrome cases. Lesions typically occur as firm and painful papules and nodules with a violaceous, erythematous and edematous appearance, affecting the extremities and trunk, and less frequently the face [37–39]. In 24% of patients, these lesions may be pruritic [37].

As in the discussed patient, arthralgia is common in VEXAS and reported in up to 28% of cases; about 58% present with non-erosive arthritis [9].

Within the first two years after onset of inflammatory symptoms venous thrombosis may occur in up to 56% of patients, whereas arterial thrombosis occurs in less than 10% of cases [40, 41].

Management of VEXAS syndrome is often challenging because of the multiorgan involvement, heterogeneity in presentation, frequent association with hematologic malignancies, dependence on glucocorticoids and refractoriness to immunosuppressive therapies [9]. Currently, there is no standardized treatment algorithm available; treatment recommendations are based on a small number of studies and case series.

Glucocorticoids at doses of 20–40 mg/day of prednisone are the most effective anti-inflammatory therapy for patients with VEXAS syndrome. As high-dose glucocorticoids are only a temporary option, and it is difficult to reduce the dosage below 10–20 mg/day due to a dose-dependent effect, most patients receive steroid-sparing agents in addition to glucocorticoids [42].

Therapeutic options include biological drugs such as IL‑1 inhibitors (anakinra and canakinumab) [10], IL‑6 inhibitors (tocilizumab) [43, 44] and TNFα blockers (infliximab, etanercept, golimumab, and adalimumab) [45, 46]; however, the effectiveness of these therapeutic approaches is individual and administration of the drugs is often discontinued due to recurrence of symptoms. Other biologicals including anti-CD20 (rituximab), anti-IL-17 (secukinumab) and anti-IL-12/IL-23 (ustekinumab) are ineffective in the treatment of VEXAS [45]; however, treatment with Janus kinase inhibitors, specifically ruxolitinib, has been associated with a response after the first month of therapy, including skin improvement, normalization of CRP levels and glucocorticoid dose reduction [9, 10, 42, 47]. According to a multicenter retrospective study evaluating the efficacy of targeted therapies in VEXAS patients, ruxolitinib and IL‑6 inhibitors are most effective with an overall response rate of 30% and 26%, respectively, at 6 months [48].

Azacitidine and decitabine, two hypomethylating agents, have also been used for the treatment of VEXAS patients with and without MDS. Data show that azacitidine is effective in the management of VEXAS syndrome leading to the improvement of inflammatory symptoms, reduction in glucocorticoid and transfusion requirements, hematologic response, normalization of the bone marrow disturbance and near-complete eradication of mutated clones [10, 45, 49–51].

Today, allogeneic hematopoietic stem cell transplantation, which aims to eradicate all cells with the *UBA1* mutation, is the sole potential curative therapy for patients with VEXAS syndrome; however, it is associated with high morbidity and mortality due to the risk of developing graft-versus-host disease and infectious complications. Therefore, this therapeutic approach is only appropriate for strictly selected patients [9, 47].

Conventional immunosuppressants such as methotrexate, cyclophosphamide, azathioprine and mycophenolate mofetil are not effective in the treatment of VEXAS syndrome [45, 52, 53]. The therapeutic benefit of immunomodulatory drugs such as hydroxychloroquine, dapsone, colchicine, lenalidomide, bortezomib and abatacept is currently unclear [10, 40, 47, 54].

The treatment of the discussed patient included glucocorticoids and ruxolitinib. In further course, a good clinical response to this therapy was evident after 8 weeks. If the patient is not in full remission and free of symptoms after 3–6 months of treatment, we plan to initiate therapy with azacitidine, which will hopefully lead to a molecular response with a substantial reduction or eradication of the *UBA1*-mutated clone [50].

### Dr. G.J. Krejs:

Data about the incidence and prevalence of VEXAS syndrome are scarcely available. So far, only one study investigated the prevalence of the syndrome based on DNA analysis from 163,096 samples of a biobank in Pennsylvania. Pathologic *UBA1 *variants were found in 11 individuals (penetrance 100%); thus, the prevalence of mutations causing VEXAS syndrome was about 1 in 14,000 unrelated individuals. In white men over 50 years, the prevalence was highest with about 1 in 4300; in women over 50 years it was about 1 in 26,200 [55].

To get more information on this disease, registries collecting patient data are increasingly being established. The French registry currently contains about 300 cases [57]. In Austria, no registry exists so far. According to Dr. Klaus Hackner from the Austrian Society of Pulmonary Medicine, five cases of VEXAS syndrome have been documented to this date in Austria.

### Dr. N. Kneidinger:

This is an instructive case of VEXAS syndrome, which is a challenging diagnosis because of the multiorgan involvement and heterogeneity in clinical presentation. Clinicians should consider this diagnosis whenever a patient presents with recurrent fever, hematologic, rheumatologic and dermatologic manifestations and markedly elevated inflammation parameters. A rapid response to glucocorticoids is also typical.

## Final diagnosis

VEXAS syndrome
